# A new species of hagfish, *Eptatretus wandoensis* sp. nov. (Agnatha, Myxinidae), from the southwestern Sea of Korea

**DOI:** 10.3897/zookeys.926.48745

**Published:** 2020-04-13

**Authors:** Young Sun Song, Jin-Koo Kim

**Affiliations:** 1 Department of Marine Biology, Pukyong National University, Busan, 48513, Korea Pukyong National University Busan South Korea

**Keywords:** mitochondrial DNA, morphology, myxinid, Myxiniformes, Northwest Pacific, taxonomy

## Abstract

Four specimens of the five-gilled white mid-dorsal line hagfish, *Eptatretus wandoensis***sp. nov.** were recently collected from the southwestern Sea of Korea (Wando). This new species has five pairs of gill apertures, 14–18 prebranchial slime pores, 4 branchial slime pores, a dark brown back with a white mid-dorsal line and a white belly. These hagfish are similar to *Eptatretus burgeri* and *Eptatretus minor* in having a white mid-dorsal line, but can be readily distinguished by the numbers of gill apertures (5 vs. 6–7), gill pouches (5 vs. 6), and prebranchial slime pores (14–18 vs. > 18), as well as the body color (dark brown back vs. gray or brown pale). In terms of genetic differences, *Eptatretus wandoensis* could be clearly distinguished from *E. burgeri* (0.9% in 16S rRNA and 8.5% in cytochrome c oxidase subunit I sequences) and *E. minor* (4.5% and 13.9%).

## Introduction

Myxinidae (hagfishes) are currently classified into six genera and 81 species worldwide (Fernholm et al. 2013; Froese and Pauly 2019). They are characterized by an eel-like body shape and 1–16 pairs of gill apertures and gill pouches; however, they have no jaws, eyes, or fins (Fernholm 1998). Recent research using morphological and molecular characteristics revealed that hagfishes comprise three subfamilies: Eptatretinae, Myxininae, and Rubicundinae (Fernholm et al. 2013). There have been several unresolved issues regarding the number of recognized genera in the subfamily Eptatretinae; however, its genera were recently reorganized taxonomically based on morphological and molecular data (Fernholm et al. 2013; Song 2019). Therefore, Eptatretinae currently includes a single genus, *Eptatretus*, which is characterized by the presence of more than two pairs of gill apertures; notably, *Eptatretus* is the most species-rich myxinid genus, currently comprising 51 valid species in the northwestern Pacific Ocean (e.g., Korea, Taiwan, and Japan) and coastal waters around Asia (e.g., China, Philippines, and Vietnam) (Froese and Pauly 2019). Surveys of the deep sea and other hard-to-reach areas using special-purpose submarines are increasingly revealing new or cryptic species worldwide (Fernholm and Quattrini 2008; Mincarone and Fernholm 2010; Zintzen et al. 2015). Based on examinations of both morphological and genetic characteristics of hagfish specimens from the southwestern Sea of Korea, we herein describe a new species, *Eptatretus wandoensis* sp. nov., and compare it with other members of the *Eptatretus* genus in around northeastern Asia.

## Materials and methods

**Figure 1.**
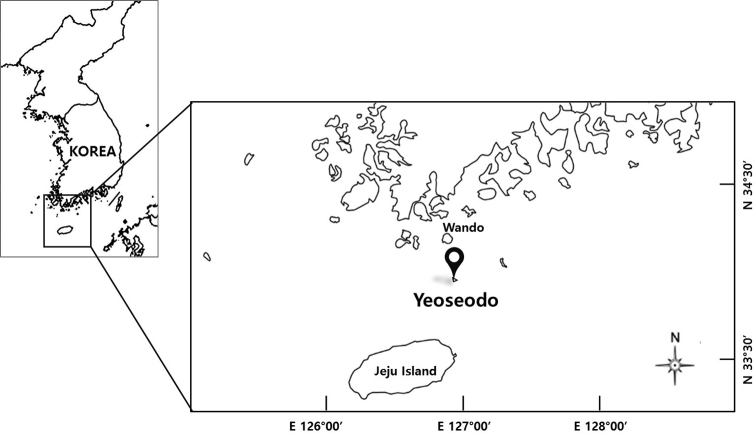
Sampling location of *Eptatretus wandoensis* sp. nov. in Korea.

We obtained four specimens (202.0–292.0 mm total length) from Yeoseo-ri, Wando-gun in Korean waters in 2018, caught by fishing trapping and bought to the fish markets (Fig. 1). The specimens have been deposited in the Marine Fish Resource Bank of Korea (MFRBK) at Pukyong National University (PKU), Busan-si, Korea. We performed morphological and molecular analyses to clarify their taxonomic status, the former based on a total of 11 counts and 13 measurements. Morphological methods and terminology followed Fernholm and Hubbs (1981) and Wisner and McMillan (1988). Each body part was measured to the nearest 0.1 mm using digital Vernier calipers, and the data were converted to percentages of the total length (TL). We counted the numbers of anterior (outer) unicusps (AUC), posterior (inner) unicusps (PUC), multicusps (= fused cusps), and total cusps according to Fernholm (1998), using a stereomicroscope (SZX-16; Olympus, Tokyo, Japan). Images were analyzed using an image analyzer (Shinhan Active Measure; Shinhan Scientific Optics, Seoul, Korea), and features were sketched using a camera lucida (SZX-DA; Olympus). We examined the anatomical characters such as the arrangement between gill pouch (GP) and efferent branchial duct (EBD). The terminology of anatomical structures followed Mok and McMillan (2004): afferent branchial arteries (ABA), efferent branchial artery (EBA), ventral aorta (VA), medial section of ventral artery (MVA), and side branchial artery (SBA).We examined (and added to) the morphological description of nasal-sinus papillae following Mok (2001) and Zintzen et al. (2015).

To compare molecular characters, total genomic DNA was extracted from the muscle tissues using 10% Chelex 100 resin (Bio-Rad, Hercules, CA) and PCR was then performed for mitochondrial DNA 16S ribosomal RNA (16S rRNA) and cytochrome c oxidase subunit I (COI), using an MJ Mini Thermal Cycler PTC-1148 (Bio-Rad) in mixtures consisting 1 μL of genomic DNA, 2 μL of 10× PCR buffer, 1.6 μL of 2.5 mM dNTPs, 0.5 μL of each primer, 0.1 μL of TaKaRa EX-*Taq* polymerase (TaKaRa Bio Inc., Kyoto, Japan), and distilled water to bring the final volume to 20 μL. PCR products were amplified using universal primers: VF2-F (5’-TCA ACC AAC CAC AAA GAC ATT GGC AC-3’) and FishR2-R (5’-ACT TCA GGG TGA CCG AAG AAT CAG AA-3’) designed by Ward et al. (2005) and 16SAR-L (5’-CGC CTG TTT ATC AAA AAC AT-3’) and 16SBR-H (5’-CCG GTC TGA ACT CAG ATC ACG T-3’) designed by Ivanova et al. (2007). The PCR profiles for the COI and 16S rRNA region consisted of initial denaturation at 95 °C for 5 min, followed by 35 cycles of denaturation at 95 °C for 1 min, annealing at 54 °C for 1 min (annealing at 50 °C in 16S rRNA), extension at 72 °C for 1 min, and a final extension at 72 °C for 5 min. The PCR products were purified using a Davinch™ PCR Purification Kit (Davinch-K Co., Ltd., Seoul, Korea). The DNA was sequenced with an Applied Biosystems ABI 3730XL sequencer (Applied Biosystems, Foster City, CA) using an ABI PRISM BigDye Terminator Cycle Sequencing Ready Reaction Kit v3.1 (Applied Biosystems). We compared our molecular data with those of the mtDNA 16S rRNA and COI sequences from various hagfish species obtained from the National Center for Biotechnology Information. Sequences were aligned using ClustalW (Thompson et al. 1994) in BioEdit version 7 (Hall 1999). The genetic divergences were calculated using the Kimura 2-parameter (K2P) (Kimura 1980) model with Mega 6 (Tamura et al. 2013). Phylogenetic trees were constructed using the neighbor-joining (NJ) method (Saitou and Nei 1987) in Mega 6 (Tamura et al. 2013), with confidence assessed based on 1000 bootstrap replications. For molecular comparison, we further analyzed the COI and 16S rRNA sequences of the *Eptatretus* species, the other hagfish specie obtained from the GenBank database. The new species sequences of each regions have been deposited with GenBank (PKU 62167, MT002683; PKU 62169, MT002684; PKU 62171, MT002685; PKU 62173, MT002686 in 16S rRNA, and PKU 62171, MT002967 in COI).

## Taxonomy

### Eptatretus wandoensis sp. nov.

Taxon classificationAnimaliaMyxiniformesMyxinidae

39B19787-0894-5335-8F34-03B9F3CE2DD3

http://zoobank.org/9C6CA8CC-BC42-48E2-87EE-47702CC46D49

**Figure 2.**
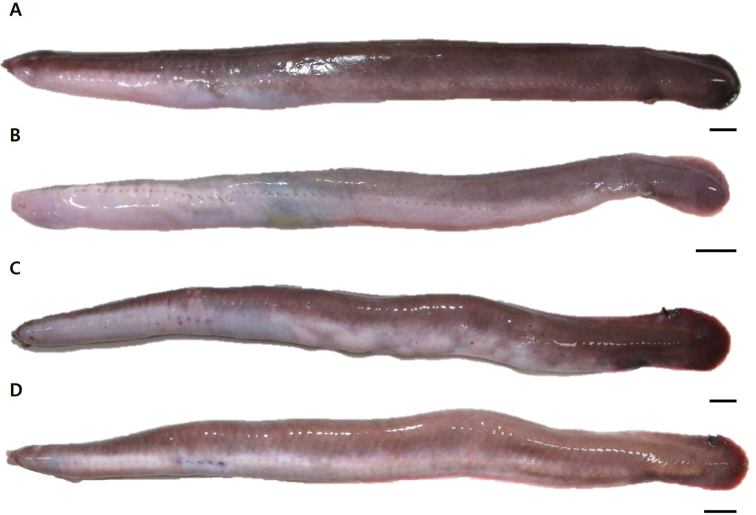
Overall view of *Eptatretus wandoensis* sp. nov., **A** holotype, PKU 62167, 292.0 mm in total length (TL) **B** paratype, PKU 62169, 202.0 mm TL**C** paratype, PKU 62171, 290.0 mm TL**D** paratype, PKU 62173, 275.0 mm TL, photographed prior to preservation. Scale bars: 1 mm.

**Figure 3.**
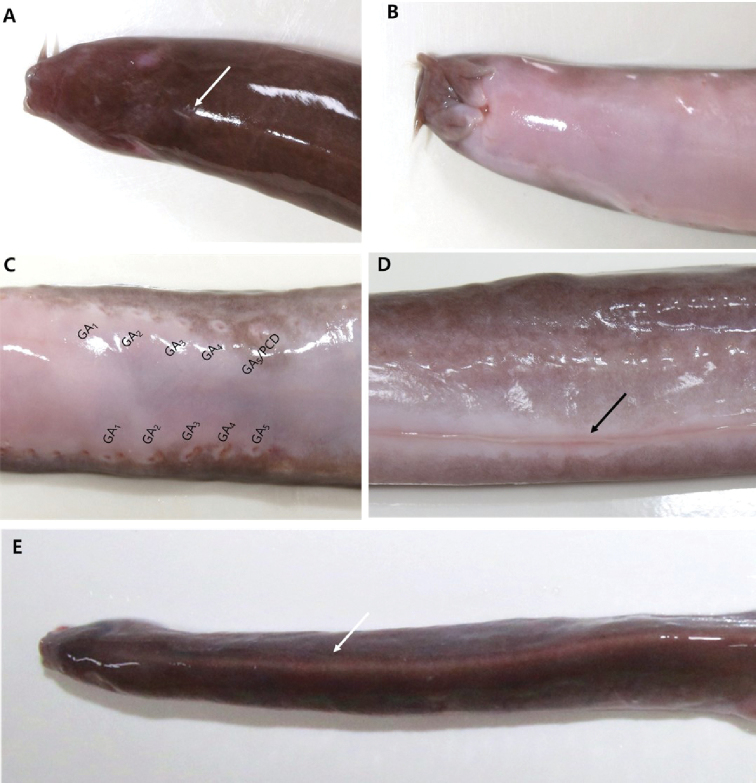
*Eptatretus wandoensis* sp. nov. PKU 62167, prior to preservation **A** head dorsal view, white arrow indicates a white mid-dorsal line **B** head ventral view **C** gill apertures (GA) and pharyngocutaneous duct (PCD), note the location of GA and PCD**D** ventral view of body, black arrow indicates the ventral fin-fold (VFF) **E** dorsal region of body with a white mid-dorsal line.

**Figure 4.**
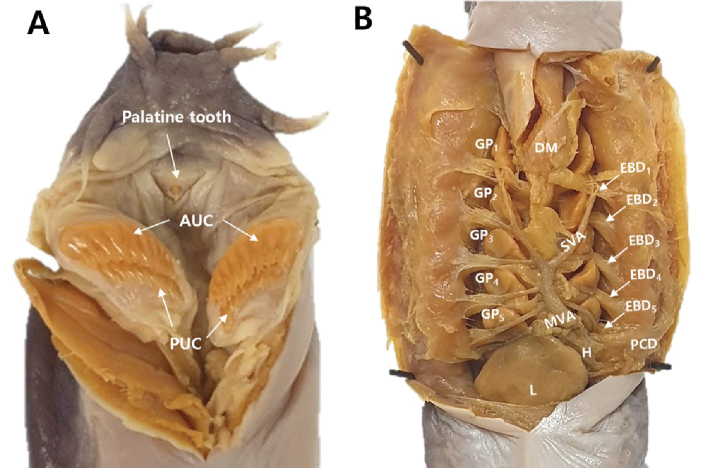
Anatomical morphology of *Eptatretus wandoensis* sp. nov. PKU 62167. **A** palatine tooth, anterior unicusps (AUC) and posterior unicusps (PUC) **B** branchial region dissected, gill pouches (GP), median ventral aorta (MVA), separated ventral aorta (SVA), efferent branchial duct (EBD), pharyngocutaneous duct (PCD), dental muscle (DM), liver (L), and heart (H).

**Figure 5.**
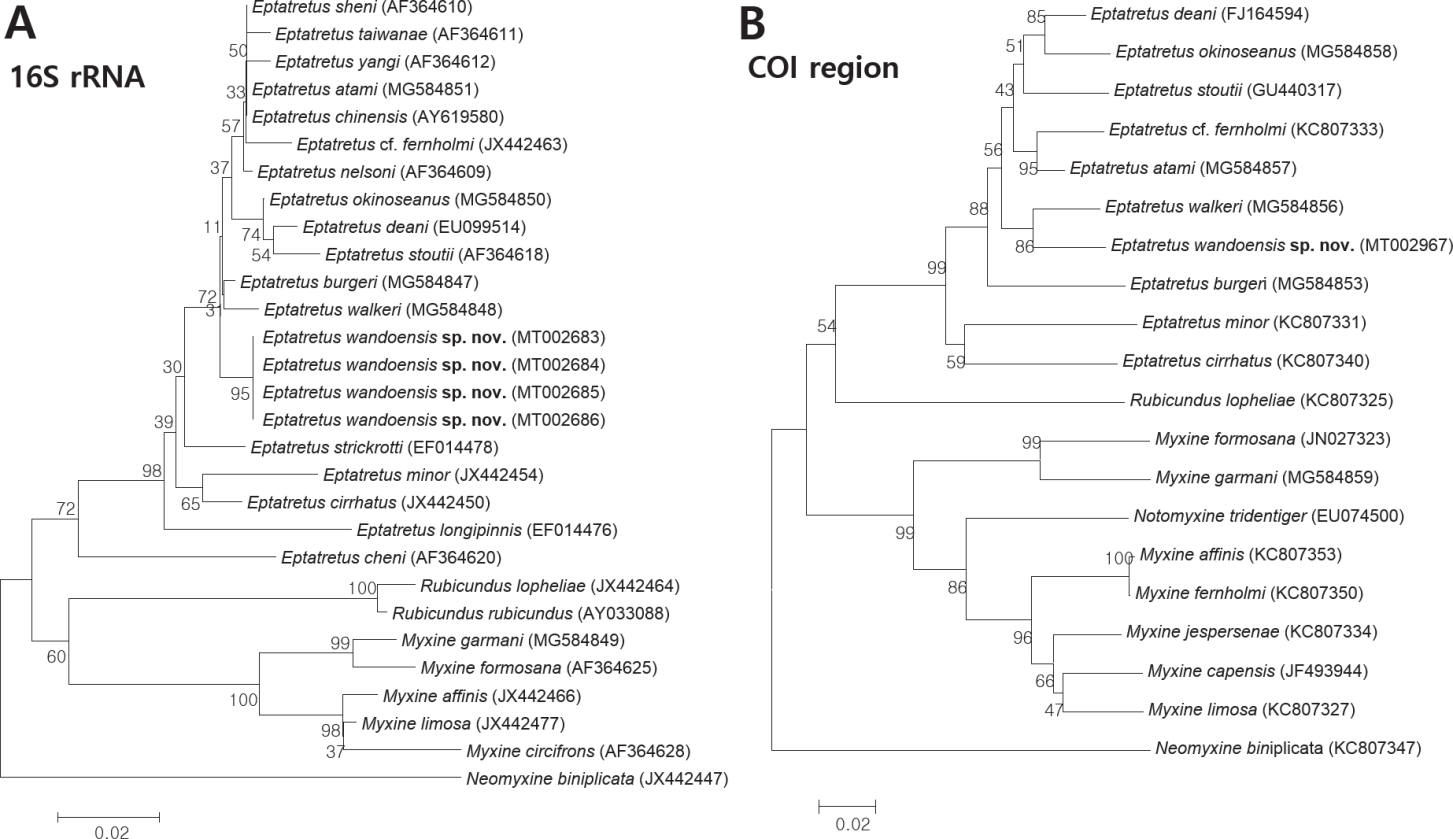
Phylogenetic tree of hagfishes based on mitochondrial DNA sequences, constructed with the Neighbor-joining (NJ) method using Kimura 2-parameter distances **A** mitochondrial DNA 16S rRNA sequences **B** mitochondrial DNA cytochrome c oxidase subunit I (COI) sequences. Numbers above tree branches are bootstrap values based on 1000 replicates. Scale bar represent nucleotide substitutions per site.

Figures 1 , 2 , 3 , 4 , 5 , Table 1 New English name: Five-gilled white mid-dorsal line hagfish; new Korean name: Huin-jul-wae-meok-jang-eo

#### Type locality.

The coast of Yeoseo-do (southwestern Sea of Korea): Yeoseo-ri, Cheongsan-myeon, Wando-gun, Jeollanam-do, Republic of Korea, 33°59'56.5"N, 126°53'57.0"E, caught by fishing traps, 60–80 m (Fig. 1).

#### Holotype.

PKU 62167, 292.0 mm TL, Yeoseo-ri, Cheongsan-myeon, Wando-gun, Jeollanam-do, Republic of Korea, 33°59'56.5"N, 126°53'57.0"E, caught by fishing traps, 60–80 m, 26 Jun 2018.

#### Paratypes.

PKU 62169 (1 specimen), 202.0 mm TL, Yeoseo-ri, Cheongsan-myeon, Wando-gun, Jeollanam-do, Republic of Korea, 33°59'56.5"N, 126°53'57.0"E, fishing trap, 60–80 m, 12 May 2018; PKU 62171, PKU 62173, 275.0–290.0 mm TL, Yeoseo-ri, Cheongsan-myeon, Wando-gun, Jeollanam-do, Republic of Korea, 33°59'56.5"N, 126°53'57.0"E, fishing trap, 60–80 m, 26 Jun 2018.

#### Diagnosis.

Gill apertures, 5; eyespots, conspicuous; fused cusps, 3/2; total cusps, 40–43; 1 GP at end of dental muscle; total slime pores, 74–82 (prebranchial, 14–18; branchial, 4; trunk, 46–49; tail, 9–11); branchial length, 5.2%–6.2% of TL; pharyngocutaneous duct confluent with last gill aperture; ventral artery splitting at approximately 3–4 GP; dorsal region with dark brown body color, ventral region with white body color; white mid-dorsal line, conspicuous.

**Table 1.** Morphometric and meristic measurements of *Eptatretus wandoensis* sp. nov., and congeneric species with five gill apertures (*E. cheni*, *E. nelsoni* and *E. yangi*) and white mid-dorsal line (*E. burgeri* and *E. minor*).

	*Eptatretus wandoensis* sp. nov.		E. cheni*	E. nelsoni*	E. yangi*	E. burgeri*	E. minor**
	Holotype	Paratypes (3)					
Gill aperture (GA)	5	5	5	5	5	6–7	6
Gill pouch (GP)	5	5	5	5	5	6	6
NSP	absent	absent	absent	absent	absent	absent	paired
**Cusps**							
MUC (multi)	3/2	3/2	3/3	3/2	3/2	3/2	3/3
AUC (outer)	7	7–8	9–11	5–8	5–8	6–8	8–11
PUC (inner)	8	8–9	9–10	5–8	6–9	7–9	8–10
Total Cusps	42	40–43	50–53	32–40	32–40	35–42	46–54
**Slime pores**							
Prebranchial	14	15–18	24–27	13–20	16–23	18–23	15–18
Branchial	4	4	0	0	0	4–5	4–6
Trunk	47	46–49	41–47	33–39	39–47	45–51	41–48
Tail	11	9–11	7–10	6–10	7–12	11–14	11–14
**Total pores**	76	74–82	75–81	57–67	68–79	81–92	74–82
**Length in % of TL**							
Prebranchial	24.7	24.4–26.3 (25.3)	33.3–35.5	30.5–32.6	29.2–32.0	25.2–29.6	20.1–25.9
Branchial	5.9	5.2–5.8 (5.5)	2.2–3.4	1.1–2.8	1.1–1.7	6.2–7.8	5.1–7.2
Trunk	56.5	54.9–59.3 (56.9)	45.9–50.8	49.5–52.6	53.2–54.9	47.6–55.0	50.6–55.9
Tail	13.4	12.8–14.0 (13.5)	13.2–16.7	15.0–18.0	12.2–15.6	13.2–17.0	13.9–18.3
Nostril to mouth	3.4	3.7–3.8 (3.8)	–	–	–	–	–
Nostril width	1.7	0.8–1.5 (1.2)	–	–	–	–	–
Nostril length	0.6	0.7–1.6 (1.1)	–	–	–	–	–
Mouth width	3.2	3.3–3.8 (3.6)	–	–	–	–	–
Pre-eyespot to nostril	4.9	4.4–5.2 (4.8)	–	–	–	–	–
**Depth in % of TL**							
w/VFF	7.5	6.9–9.7 (8.3)	8.1–9.0	15.0–15.5	6.9–10.4	4.7–8.5	7.1–11.4
Branchial region	6.3	5.6–7.7 (6.7)	–	–	–	–	–
Over caudal	7.8	7.6–9.3 (8.6)	7.6–10.2	8.9–10.1	6.5–10.0	5.1–8.5	5.3–11.6

*McMillan and Wisner (2004), **Fernholm and Hubbs (1981); Abbreviation: NSP (nasal-sinus papillae), MUC (multicusps), AUC (anterior unicusps), PUC (posterior unicusps), VFF (ventral fin-fold).

#### Description.

Body elongated; laterally compressed at trunk and strongly compressed at tail (Fig. 2). Rostrum slightly blunt and round (Fig. 3A). Nasal-sinus papilla absent. Eyespots present (Fig. 3A). Pre-eyespots shorter than branchial region (4.4%–4.9% of TL). Three pairs of barbels on head: first (1.5%–1.6% of TL) and second barbels (1.6%–1.9% of TL) nearly equal in size; third barbel is longer (2.1%–2.3% of TL) and tips of third barbels extend at the mouth (Fig. 3B). Five pairs of GP and apertures; each gill aperture arranged regularly spaced in a straight line (Fig. 3C). Teeth row comb-like, consisting of two rows with tips sharp and curved rearward (Fig. 4A); in the outer row, 3 multicusps and 7–8 unicusps; in the inner row, 2 multicusps and 8–9 unicusps; total number of cusps, 40–43. Dental muscle thick and long, posterior tip of dental muscle located in first GP (Fig. 4B). Slime pores: prebranchial, 14–18; branchial, 4; trunk, 46–49; tail, 9–11; total, 74–82. Body proportions are as follows: prebranchial length, 24.4%–26.3% of TL; branchial length, 5.2%–6.2% of TL; trunk length, 54.9%–59.3% of TL; tail length, 12.8%–14.0% of TL; cutaneous duct, 7.6%–9.3% of TL; branchial duct (with ventral fin-fold), 6.9%–9.7% of TL; and branchial duct (alone), 5.6%–7.7% of TL (Table 1). Posterior-most EBD confluent with pharyngocutaneous duct on left side, forming a larger aperture (Fig. 4B). All efferent branchial ducts are equal in length. VA consists of two SBAs and one medial section, bifurcating at approximately the third or fourth GP. First through third pairs of ABAs, which cannot be regarded as branches of the VA, branch from SBAs; however, fourth and fifth ABAs on left and right branch from the medial section of the ventral artery (Fig. 4B). Ventral fin-fold weakly developed or vestigial, beginning approximately at middle of body and extending to cloaca (Fig. 3D). Caudal fin-fold weakly developed, beginning posterior cloaca and extending around tail to dorsal surface.

Coloration when fresh: Body uniformly dark brown or purplish dorsally and white ventrally; white mid-dorsal line conspicuous, beginning from the upper region of the first prebranchial slime pore to around the tail. Eyespots conspicuous; whole barbels (rarely the tip) pale, and pale around mouth. Each gill aperture and pharyngocutaneous duct aperture with white margin; most slime pores blackish (except for tail region), tail slime pores same as surrounding color. White around cloaca; ventral fin-fold with a white line along the ventral midline; posterior margin of caudal fin pale (Fig. 2).

Coloration when preserved: Body brown to dark brown dorsally and murky white ventrally (more conspicuous than fresh specimen). Eyespots conspicuous; all slime pores surrounded by conspicuous white ring. Each gill aperture and pharyngocutaneous duct aperture conspicuous; ventral fin-fold pale; white mid-dorsal line inconspicuous.

#### Distribution.

Southwestern Sea of Korea.

#### Biology.

Attains a maximum TL of 292.0 mm (fresh specimen); this specimen is female, without mature eggs in the body cavity. A female specimen of 290.0 mm TL carries approximately 20 developing eggs, which have no terminal anchor filaments or hooks; each egg approximately 4–7 mm in diameter and 10–12 mm in length.

#### Etymology.

The specific name, *wandoensis*, refers to the type locality, in Korea.

##### Morphological comparisons

*Eptatretus wandoensis* sp. nov. is most similar to *Eptatetus burgeri* (Girard, 1855) and *Eptatretus minor* Fernholm & Hubbs, 1981 due to the presence of a light mid-dorsal line, gill apertures regularly spaced in a straight line, and EBDs of equal length. These three species differ from each other in the number of gill apertures (5 for *E. wandoensis*, compared to 6 for *E. burgeri* and *E. minor*), body color (dark brown or purplish dorsally and white ventrally for *E. wandoensis*, compared to brown for *E. burgeri* and gray/brown pale for *E. minor*), prebranchial slime pores (14–18 for *E. wandoensis*, compared to 18–23 for *E. burgeri*), total slime pores (74–82 for *E. wandoensis*, compared to 81–92 for *E. burgeri*), ventral fin-fold (weakly developed for *E. wandoensis*, compared to well developed for *E. burgeri*), multicusps (3/2 for *E. wandoensis*, compared to 3/3 for *E. minor*), total cusps (40–43 for *E. wandoensis*, compared to 46–52 for *E. minor*) (Table 1), nasal-sinus papillae (absent for *E. wandoensis*, compared to paired for *E. minor*), and eyespots (present for *E. wandoensis*, compared to absent for *E. minor*). *Eptatretus wandoensis* sp. nov. can be distinguished from *Eptatretus cheni* (Shen & Tao, 1975), *Eptatretus nelsoni* (Kuo, Huang & Mok, 1994), and *Eptatretus yangi* (Teng, 1958) by the presence of regularly spaced gill apertures in a linear (vs. irregular and crowded for *E. cheni*, *E. nelsoni*, and *E. yangi*) arrangement; equal length of all EBDs (vs. length of first efferent branchial duct notably longer than that of the most posterior efferent branchial duct); 40–43 total cusps (vs. 50–53 for *E. cheni*; 32–40 for *E. nelsoni* and *E. yangi*); 4 branchial slime pores (vs. no branchial slime pores); prebranchial length, 24.4%–26.3% of TL (vs. more than 29.0% of TL); branchial length, 5.2%–5.9% of TL (vs. less than 3.4% of TL); trunk length, 54.9–59.3% of TL (vs. less than 54.9%); eyespots conspicuous (vs. inconspicuous), and dorsal dark brown and ventral white body color (vs. brownish-grey). In comparison to *Eptatretus* species occurring in Korean and Japanese waters, this new species is well distinguished from the three most common hagfishes, *Eptatretus atami* (Dean, 1904), *Eptatretus walkeri* (McMillan & Wisner, 2004), and *Eptatretus okinoseanus* (Dean, 1904) based on the difference of gill apertures (5 in *E. wandoensis* sp. nov. vs. 6 in *E. atami* and *E. walkeri* vs. 8 in *E. okinoseanus*), branchial slime pores (4, 0-1, 0, and 6-8), and a white mid-dorsal line (present, absent, absent, and absent) (Table 2).

**Table 2.** Comparison of meristic and proportional measurements among *Eptatretus* species occurring in Korean and Japanese waters.

Characters	*E. wandoensis* sp. nov.	E. atami*	*E. walkeri*	E. okinoseanus*
Gill aperture	5	6	6	8
Gill pouch	5	6	6	8
NSP	absent	absent	absent	absent
**Cusps**				
MUC	3/2	3/3	3/2	3/2
AUC	7–8	9–10	6–9	7–10
PUC	8–9	8–10	7–9	7–10
Total	40–43	47–52	36–44	40–49
**Slime pores**				
Prebranchial	14–18	12–19	15–22	13–17
Branchial	4	0–1	0	6–8
Trunk	46–49	43–47	40–48	54–61
Tail	9–11	9–12	8–13	10–14
**Total pores**	74–82	71–78	68–79	87–97
**Length in % of TL**				
Prebranchial	24.4–26.3	26.6–30.2	24.2–39.1	19.2–22.6
Branchial	5.2–5.9	1.3–4.2	2.0–3.8	6.2–9.2
Trunk	54.9–59.3	53.9–56.1	50.8–68.6	50.4–59.4
Tail	12.8–14.0	11.1–14.2	10.7–16.1	12.7–15.5
**Depth (mm)**				
w/VFF	6.9–9.7	8.1–9.0	5.0–11.1	5.7–8.1
Over caudal	7.6–9.3	7.4–8.8	6.3–11.4	6.2–9.0

*McMillan and Wisner (2004); Abbreviation: NSP (nasal-sinus papillae), MUC (multicusps), AUC (anterior unicusps), PUC (posterior unicusps), VFF (ventral fin-fold).

##### Genetic comparisons

Differences among mtDNA sequences obtained from the holotype and paratypes of *Eptatretus wandoensis* sp. nov. were consistent with species-level divergences in other hagfish species (Fernholm et al. 2013). The phylogenetic relationships of myxinid species, inferred from neighbor-joining trees, showed large genetic distances between similar hagfish species using mtDNA 16S rRNA (477 bp) and cytochrome c oxidase subunit I (COI) (466 bp) sequences. *Eptatretus wandoensis* sp. nov. is separated from other congeneric species by high genetic divergences of 0.9%–7.5% in 16S rRNA sequences and 4.9%–13.9% in COI sequences (Fig. 5). The respective genetic distances between this species and *E. burgeri* and *E. minor* were 0.9% and 4.5% in 16S rRNA sequences and 8.5% and 13.9% in COI sequences. In addition, phylogenetic analysis of 16S rRNA sequences showed that *E. wandoensis* sp. nov. is well separated from other five-gilled hagfishes (*E. cheni*, *E. nelsoni*, and *E. yangi*), with genetic differences of 7.5%, 1.4%, and 1.6%, respectively. *Eptatretus cheni* is located at a basal position of hagfishes and well nested in the *Eptatretus* clade.

## Discussion

*Eptatretus wandoensis* sp. nov. is one of many new hagfish species recently discovered in the northwest Pacific Ocean. Thus far, six hagfish species with five gill apertures have been reported worldwide (McMillan and Wisner 2004; Kuo et al. 2010; Zintzen et al. 2015); most are included in the genus *Eptatretus* (Fernholm et al. 2013). However, three species have tubular nostrils and pink coloration; thus, they are regarded as *Rubicundus* species (Fernholm et al. 2013; Zintzen et al. 2015). This new species is the third member of the genus with a white mid-dorsal line, after *Eptatretus burgeri* and *E. minor* (Girard 1855; Fernholm and Hubbs 1981). This new species was initially confused with *E. burgeri* because it may have been considered a morphological variation of *E. burgeri*, due to the presence of five gill apertures. However, they are well distinguished by the body color, prebranchial slime pores, total slime pores, and ventral fin-fold. In addition, we found a female specimen with ripe eggs on June 26, 2018. Recent study revealed that the minimum mature size *Eptatretus burgeri* with ripe eggs is more than 500.0 mm TL (Song 2019); however, this female specimen was 290.0 mm TL. Recently, specific anatomical structures such as cusps, nasal-sinus papillae, and heart have been regarded as useful characters for clarifying interrelationship among hagfish (Mok 2001; Icardo et al. 2016a; Icardo et al. 2016b). Indeed, Mok (2001) suggested that the absence of nasal-sinus papillae may be an apomorphic character of most eptatretines. Interestingly, all three *Eptatretus* species have no nasal-sinus papillae (Song and Kim 2020), and so therefore well supports the hypothesis of Mok (2001). Phylogenetic trees indicated that the new species is sister-group to *E. walkeri* (supported by CO1 gene), but *E. walkeri* becomes the sister-group of *E. burgeri* (supported by 16S rRNA gene). Naylor and Brown (1998) mentioned that genes yielding correct results might vary among data sets and thus this discordance might be influenced by stochastic error associated with a different number of species and data sets. Kawaguchi et al (2001) suggested that taxonomic sampling and comprehensive sequencing may clarify intra- and interrelationships of fish using mitochondrial data.

In terms of geographic distribution, *Eptatretus minor* occurs in the Gulf of Mexico and Atlantic Ocean, while *E. burgeri* coexists with this new species in the same region of coastal Korea. In the comparison of depths, *Eptatretus wandoensis* sp. nov. is collected from depths between 60 to 80 m, and *E. burgeri* is known as between 5 and 270 m, and *E. minor* is known as between 300 and 400 m (Fernholm and Hubbs 1981; Moller and Jones 2007; Knapp et al. 2011; Angulo and Moral-Flores 2016). Among them, *Eptatretus minor* is deeper than the other two species. Interestingly, most Korean hagfishes tend to be distributed in quite shallow waters (within 100 m water depth) (Song 2019; Song and Kim 2020). In a recent morphological and molecular taxonomic review of *Eptatretus atami* from the coast of Japan, the specimens with 3/2 multicusps from the western coast of Honshu were identified as *E. walkeri*, whereas eastern specimens with 3/3 multicusps matched *E. atami* (Kase et al. 2017; Kitano et al. 2019). Later, Song and Kim (2020) revealed for the first time the existence of *E. walkeri* previously misidentified as *E. atami* in Korea, and confirmed that three species are currently distributed in Korea.

## Supplementary Material

XML Treatment for Eptatretus wandoensis
